# Low-level expression of HER2 and CK19 in normal peripheral blood mononuclear cells: relevance for detection of circulating tumor cells

**DOI:** 10.1186/1756-8722-1-2

**Published:** 2008-05-28

**Authors:** Fanglei You, Lisa A Roberts, S Peter Kang, Raquel A Nunes, Cinara Dias, J Dirk Iglehart, Natalie A Solomon, Paula N Friedman, Lyndsay N Harris

**Affiliations:** 1 Department of Cancer biology/Adult Oncology, Dana-Farber Cancer Institute, Boston, MA 02115, USA; 2 Abbott Molecular, Inc. 1300 E. Touhy Avenue, Des Plaines, IL 60018, USA; 3 Department of Surgery, Brigham and Women's Hospital, Boston, MA 02115, USA; 4 Section of Medical Oncology, Yale University School of Medicine/Yale Cancer Center, 333 Cedar Street, New Haven, Connecticut 06520, USA

## Abstract

**Background:**

Detection of circulating tumor cells (CTC) in the blood of cancer patients may have prognostic and predictive significance. However, background expression of 'tumor specific markers' in peripheral blood mononuclear cells (PBMC) may confound these studies. The goal of this study was to identify the origin of Cytokeratin 19 (CK19) and HER-2 signal in PBMC and suggest an approach to enhance techniques involved in detection of CTC in breast cancer patients.

**Methods:**

PBMC from healthy donors were isolated and fractionated into monocytes, lymphocytes, natural killer cells/granulocytes and epithelial populations using immunomagnetic selection and fluorescent cell-sorting for each cell type. RNA isolated from each fraction was analyzed for CK19, HER2 and Beta 2 microglobulin (B2M) using real-time qRT-PCR. Positive selection for epithelial cells and negative selection for NK/granulocytes were used in an attempt to reduce background expression of CK19 and HER2 markers.

**Results:**

In normal PBMC, CK19 was expressed in the lymphocyte population while HER-2 expression was highest in the NK/granulocyte population. Immunomagnetic selection for epithelial cells reduced background CK19 signal to a frequency of <5% in normal donors. Using negative selection, the majority (74–98%) of HER2 signal could be removed from PBMC. Positive selection methods are variably effective at reducing these background signals.

**Conclusion:**

We present a novel method to improve the specificity of the traditional method of detecting CTC by identifying the source of the background signals and reducing them by negative immunoselection. Further studies are warranted to improve sensitivity and specificity of methods of detecting CTC will prove to be useful tools for clinicians in determining prognosis and monitoring treatment responses of breast cancer patients.

## Background

The presence of circulating tumor cells (CTC) in peripheral blood and disseminated tumor cells (DTC) in bone marrow has been associated with negative clinical outcomes in numerous studies [1-4]. The capacity to detect CTC in the peripheral blood of cancer patients may provide a unique tool to determine prognosis and monitor for recurrence of breast cancer [5-7]. Unlike currently available tumor markers, the advantage of CTC might be the ability to characterize tumor phenotype *ex vivo*, providing what could be considered as a 'virtual biopsy' of tumor tissue.

While the study of CTC in circulation is an active area of research, many challenges remain to accurately characterize these cells. Firstly, tumor cells in circulation are infrequent, ranging from 1/105 to 1/107 peripheral blood mononuclear cells (PBMC), even in patients with metastatic tumors[5]. In an effort to improve sensitivity, analysis of gene expression using reverse transcription polymerase chain reaction (RT-PCR) has been employed for detection of micrometastases. While these methods have increased sensitivity, and allow the detection of as few as one epithelial cell in 107 mononuclear blood cells, specificity remains an important problem [5]. One of the factors that compromises the specificity of RT-PCR methods in detecting micrometastases is the background expression of 'tumor markers' in normal peripheral blood. Understanding the origin of background and developing methods to selectively eliminate it is a critical step to improving the specificity of the RT-PCR method.

The goal of this study is to identify the source of background signals for Cytokeratin 19 (CK19) and HER-2 in PBMC and propose an approach to reduce the cells contributing to the background to improve the specificity of a currently available and sensitive method of detecting CTC. We measured CK19 and HER2 in PBMC using quantitative, real-time RT-PCR after immunomagnetic selection for epithelial cells using BerEP4 antibody. We found that CK19 signal was occasionally observed in the peripheral blood of normal controls, and that the HER2 signal was frequently present in the peripheral blood of both normal controls and breast cancer patients. In addition, the HER2 signal seen in the blood of breast cancer patients was not restricted to patients with HER2 positive tumors. To better understand the source of the HER2 and CK19 signals in peripheral blood, we isolated subpopulations from the PBMC fraction and characterized them for HER2 and CK19. Understanding the biology of the background expression of tumor markers will be instrumental in development of more specific methods to detect CTC.

## Materials and methods

### Metastatic Breast Cancer Patient Blood samples

Blood samples were obtained from 120 untreated metastatic breast cancer patients on an IRB-approved trial for the study of biomarkers in blood of breast cancer patients. HER2 levels were characterized by immunohistochemistry (DAKO Herceptest^®^) on primary tumors from these patients and considered positive if the tumor showed 3+ membrane staining.

### Isolation of PBMC from Whole Blood

Blood was collected from each human subject in 8 ml CPT Vacutainer tubes (BD Biosciences) and centrifuged within 2 hours of a blood draw at 2800 rpm for 30 minutes at room temperature in a Beckman CS-6R with a swinging bucket rotor. The cells above the gel plug were resuspended in the plasma layer, washed once in 2% FBS, 0.6% Sodium citrate, DPBS (without Ca^++^/Mg^++^) and centrifuged at 1200 rpm for 10 minutes to obtain the PBMC fraction.

### Serial Immunomagnetic Positive Selection

Thirty-two milliliters of blood was collected in 4 CPT blood collection tubes from each of 4 healthy human subjects under an approved IRB protocol. For each subject, the PBMC fraction from one tube was resuspended in 1 mL 1% FBS, 0.6% Sodium citrate, DPBS (without Ca^++^/Mg^++^) and subjected to immunomagnetic selection with Dynal M450 Sheep anti-mouse magnetic particles coated with 40 μg/mL BerEP4 antibody (Dako) per manufacturer's instructions.

Two tubes from each subject were resuspended in 2 mL 0.1% BSA, 1 mM EDTA, DPBS (without Ca^++^/Mg^++^) and then subjected to serial immunomagnetic selection. Briefly, Dynal M450 Sheep anti-mouse magnetic particles were coated with 40 μg/mL α-CD3 antibody (clone UCHT1, Dako), α-CD19 antibody (clone HD37, Dako), α-CD14 antibody (clone M5E2, Pharminagen) or α-CD16 antibody (clone 3G8, Pharminagen). Each PBMC aliquot was incubated with 250 μL α-CD3 antibody and 50 μL α-CD19 coated particles for 1 hour at 2–8°C. The magnetic beads were collected and the supernatants were transferred to a new tube. The supernatants underwent serial immunomagnetic selection with 100 μL α-CD14 coated magnetic particles followed by 25 μL α-CD16 coated microparticles. Each α-CD positively selected population was washed 3× with 2 mL BSA/EDTA buffer before proceeding to RNA isolation. Cell selection efficiency and specificity was determined by obtaining cell profiles on the starting PBMC sample and each transferred supernatant using the Hematology Analyzer Abbott CellDyn 3000. The PBMC fraction from one tube per subject underwent RNA isolation and served as a total RNA (unselected) control.

### Immunomagnetic Selection of Individual PBMC Subpopulations

Seven CPT tubes (56 mL) were collected from each of 4 healthy human subjects under an IRB-approved protocol and the PBMC fractions were isolated. Immunomagnetic selection was performed using the protocol listed above. Each tube was selected independently (BerEP4, α-CD3, α-CD19, α-CD14, α-CD16, or α-CD56 (25 μL)). The supernatants from these 6 tubes and the 7^th^, unselected, tube, were gently spun down and the cells underwent RNA isolation followed by HER-2, CK19 and B2M RNA quantitation using the Real Time RT-PCR Assays.

### RNA isolation and Real Time RT-PCR

RNA was isolated from each positive and negative selected cell sample using the RNeasy^® ^mini RNA isolation kit (Qiagen) and eluted in 50 μL per the manufacturer's instructions. Real-time RT-PCR for HER-2 was performed with 5 μl of RNA template and the Promega Access Amplification kit (Promega Inc. Madison, WI) using 1.5 mM MgSO_4 _and 200 nM HER-2 primers, 300 nM HER-2 Taqman probe (Table 1: Sequence of primers used in the paper). Real time RT-PCR was performed on a BioRad iCycler with the following cycling conditions: 1 cycle at 48°C for 45 minutes, 1 cycle at 95°C for 1 minute, 40 cycles of 96°C for 1 second, 66°C for 30 seconds.

**Table 1:** Sequence of primers used in the paper

Primer/Probe	Sequence
Her-2 Forward Primer	5' CCCAACCAGGCGCAGAT 3'
Her-2 Reverse Primer	5' AGGGATCCAGATGCCCTTGTA 3'
Her-2 Taqman Probe	5' 6FAM-CGCCAGATCCAAGCACCTTCACCTT-TAMRA 3'
CK19 Forward Primer	5' CCGCGACTACAGCCACTACTACAC 3'
CK19 Reverse Primer	5' GAGCCTGTTCCGTCTCAAA 3'
CK19 FAM Beacon Probe	5' FAM-CGTGGTGCCACCATTGAGAACTCCAGGACCACG-BHQ1 3'

For the CK19/B2M Duplex assay, 5 μl of RNA template was added to 45 μl Master Mix (Promega Access Amplification kit), using 2.0 mM MgSO_4 _and 200 nM B2M Forward and Reverse primer, 300 nM B2M Vic Beacon, 250 nM CK19 Forward primer, 500 nM CK19 Reverse primer and 300 nM CK19 FAM Beacon probe (Table 1: Sequence of primers used in the paper). Individual RUO CK19 and B2M primer/probe mixes are now available (Abbott Molecular, Inc., Des Plaines, IL). Real time RT-PCR was performed on an ABI Prism 7000 Real Time Thermalcycler with the following cycling condition: 1 cycle at 48°C for 45 minutes, 1 cycle at 94°C for 1 minute, 5 cycles of 94°C for 15 seconds, 63°C for 30 seconds followed by 40 cycles of 94°C for 1 second, 62°C for 30 seconds, and 50°C for 30 seconds.

HER-2 and CK19 quantities were calculated using an MDA-MB-361 breast cancer cell line standard curve and expressed as MDA-MB-361 cell equivalents of RNA (ce). B2M quantitation was determined from a normal human PBMC pool RNA standard curve.

### CK19 detection by the Abbott LCx method

Amplification was performed using unit dose vials containing buffer, nucleotides and a thermostable polymerase with reverse transcriptase activity. Prior to amplification, the oligonucleotide mix, Mn^++ ^and 5 μL of RNA were added to the unit dose vial. Thermal cycling conditions were as follows: incubation at 60° for 60 minutes, then 94° for 40 seconds and 58° for 1 minute for 45 cycles. After cycling was complete, the temperature was increased above the melting point of the amplification product and quickly lowered to 12°C, to allow the detection probe present in the mix to anneal to dissociated product strands and generate a detectable amplicon-probe complex. Microparticle Enzyme Immunoassay (MEIA) detection using the LCx^® ^Analyzer (Abbott Laboratories) was performed as previously described,[8] and the results are reported as counts/sec/sec (c/s/s).

### HER2 RT-PCR Assay Sensitivity

Approximately 1000, 500, or 100 SKBR3, MDA-MB-361, MDA-MB-453 or MCF-7 Cells (ATCC) were spiked into aliquots of 1 × 10^7 ^PBMC (Normal donor leukopak) and subjected to immunomagnetic selection with BerEP4 antibody coated beads and RNA isolation per the protocols above. One-tenth of each RNA sample was analyzed by the HER2 qRT-PCR assay. One and 0.1 cell equivalent samples were derived from 10 and 100 fold dilutions of the 100 cell spiked RNA samples.

### Cell Sorting by Flow Cytometry

The PBMC fraction was isolated from 6 CPT Vacutainer blood tubes collected from one healthy human subject per the protocol above. The PBMC were washed a total of 3 times, pooled and resuspended to 2.0 × 10^7 ^cells/mL in RPMI 1640 media. 3.5 × 10^7 ^PBMC were incubated with 704 μL of α-CD3-Cy5, α-CD19-APC, α-CD16-FITC, and α-CD14-PE (Pharminagen) in the dark for 30 minutes on ice. The labeled cells were washed once in RPMI media, filtered through a 35 μm mesh filter tube with strainer cap (Falcon) and then placed in the cell sorter (MoFLO, DakoCytomation Ft. Collins, CO). Two-thirds of the sample was sorted for α-CD16-FITC and α-CD14-PE while the remaining third of the sample was sorted for α-CD19-APC/α-CD3-Cy5 and α-CD16-FITC. Two million PBMC were incubated with mouse isotype control antibodies labeled with each fluorophore. These samples served as negative controls to adjust the cell sorter instrument settings. The isolated cells were characterized for purity after sorting and then spun down and resuspended in RNeasy lysis buffer for subsequent RNA isolation.

## Results

**Figure 1:**
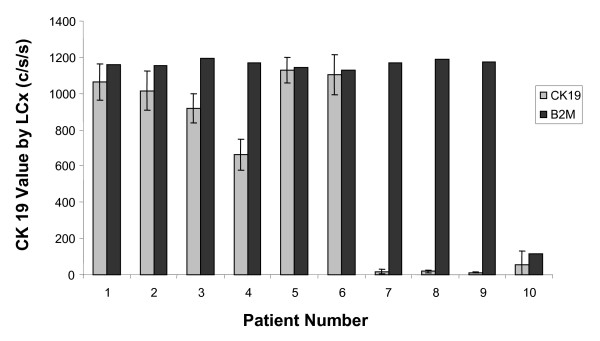
**Detection of Cytokeratin 19 (CK19) by LCx in metastatic breast cancer patients**. Semi-quantitative RT-PCR for CK19 and Beta2 microglobulin (B2M) was performed on PBMC after BerEP4 immunomagnetic selection for malignant epithelial cells from 10 patients with untreated metastatic breast cancer. CK19 assays were run in duplicate on two separate occasions with the average from each sample shown using error bars. B2M assays were run once per sample as previous experiments have shown the CV of duplicates to be <2%. The change in fluorescent energy serves as the reported value expressed in counts/sec/sec (c/s/s).

Semi-quantitative RT-PCR LCx assays for CK19 and B2M were developed to detect epithelial cells from the peripheral blood of patients with metastatic breast cancer. Figure 1 depicts a representative sample of 10 patients with metastatic breast cancer prior to adjuvant treatment (See Figure 1).

**Figure 2:**
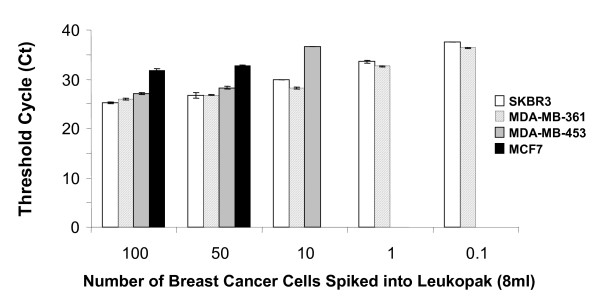
**Titration of cell lines for HER2 signal**. To test the sensitivity of HER2 detection in blood, real time quantitative RT-PCR was performed on RNA isolated from mock positive controls of 8 ml of leukopak cells spiked with serial dilutions of breast cancer cell lines with varying levels of HER2 expression. The control samples were immunomagnetically selected with BerEP4 antibody prior to RNA isolation. Error bars represent the standard deviation of duplicate PCR reactions. If a sample never crossed the threshold, it is plotted as zero on this graph.

Sample 10 demonstrates the utility of the B2M assay to assess RNA integrity as low B2M signal indicates that the RNA is not adequate and the CK19 result cannot be interpreted. These assays were highly specific with CK19 signal present in 50–60% of metastatic breast cancer patients (n = 120) and <5% of normal donors (n = 75); however this method was only semi-quantitative. To better characterize circulating tumor cells, we developed quantitative RT-PCR assays for CK19 and HER2 mRNA. To test the sensitivity of HER2 detection, real time quantitative RT-PCR was performed on BerEP4 immunomagnetic selected leukopak blood spiked with serial dilutions of breast cancer cell lines with varying levels of HER2 amplification [9,10]. In MDA-MB-361 and SK-BR3 cells, with relatively high HER2 expression, real time RT-PCR could detect 0.1 cell equivalent (ce) spiked into 8 mL of peripheral blood. The detection limit increased to 10 ce and 50 ce per 8 mL in cell lines expressing intermediate and low levels of HER2 (MDA-MB-453 and MCF7 respectively) (See Figure 2).

The sensitivity of the CK19 assay, as tested by dilutions of the cell line RNAs, was approximately 0.01 ce for each cell line (data not shown). Using this method in healthy control samples subjected to immunomagnetic selection with BerEP4, we verified that HER2 was consistently expressed, although at a lower level than in spiked samples.

**Figure 3:**
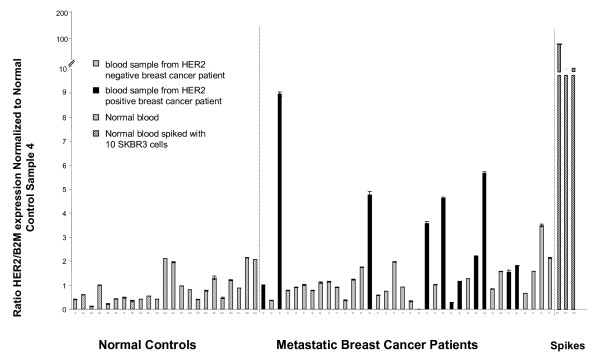
**HER2 signal after BerEP4 selection in normal donors, patient samples and SKBR3 spiked normal blood samples**. Blood samples from 36 metastatic breast cancer patients, 23 normal donors and three normal donor samples spiked with 10 SKBR3 human breast cancer cells were subjected to BerEP4 immunomagnetic enrichment for epithelial cells. After RNA extraction from the positively selected cellular fraction, HER2 and B2M expression were quantitated by real time qRT-PCR. The standard curve was obtained by serial dilution of DNA from a HER2 positive breast cancer cell line (BT474). HER2 relative expression per sample was calculated by obtaining the HER2 value/B2M value ratio for each sample and then normalizing against the HER2/B2M ratio of Normal Control Sample N4, as it represented the median value for HER2 in normal samples. Error bars represent the standard deviation of triplicate reactions. Normal controls are depicted by cross hatched mark. Samples from breast cancer patients are depicted in grey bars (HER2 negative tumors) and black bars (HER2 positive tumors).

We then further explored our ability to detect HER2 expressing CTC from patients and evaluated whether a cut-off in HER2 expression could be established between healthy controls/HER2 negative patients and HER2 positive patients with CTC. In this experiment, we subjected peripheral blood samples from 36 patients with metastatic breast cancer and 23 normal donors to immunomagnetic enrichment for epithelial cells using the BerEP4 antibody. After RNA extraction from the positively selected cellular fraction, real time RT-PCR was performed to detect HER2 mRNA in these samples. While there was a distinct difference in the amount of HER2 signal in metastatic patients compared with normal controls, significant overlap was seen between these populations (See Figure 3) In addition, patients whose tumors were HER2 positive (black bars), were more likely to have a positive signal for HER2 but considerable overlap was seen between HER2 positive and negative tumors. This suggests that the HER2 signal in peripheral blood might not be specific for epithelial cells. Immunomagnetic selection typically decreases the PBMC in the sample by 1000-fold or more. However, in instances where the enrichment is less than 1000-fold, it is possible to see some background CK19 signal in samples from normal subjects (data not shown). The levels of signal are lower than for HER2, but still may confound the interpretation of the CK19 signal.

**Figure 4:**
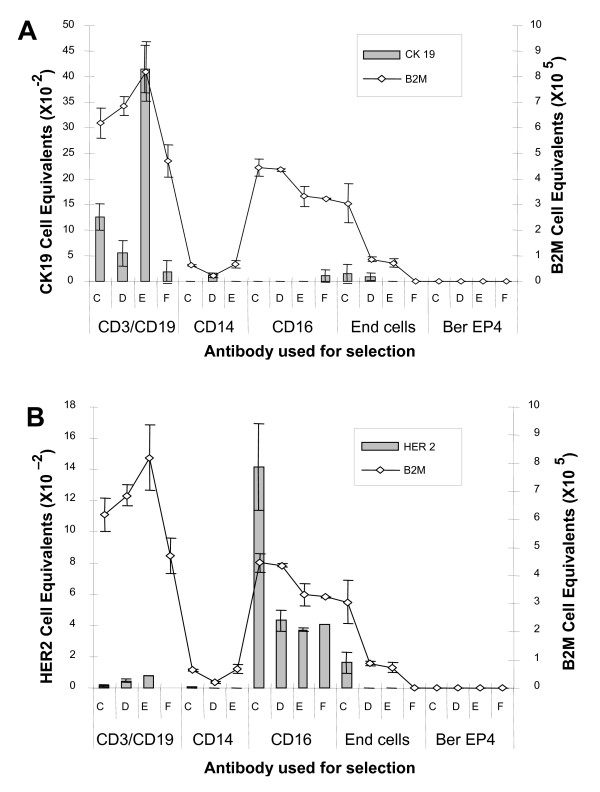
**CK19 and HER2 RNA expression from serial immunomagnetic selection of peripheral blood mononuclear cells (PBMC) from 4 normal subjects**. Subpopulations of PBMC from 4 normal subjects (C, D, E, F) were isolated by serial immunomagnetic selection with CD14 (monocytes), CD3/CD19 (lymphocytes), and CD16 (natural killer cells/granulocytes). An additional PBMC sample from each subject underwent immunomagnetic selection with BerEP4 (epithelial cells) as a negative control. MDA-MB-361 RNA was used for standard curves when detecting CK19 and HER2. RNA from normal leukocytes was used for standard curve for B2M detection. End cells: cells remaining after the serial selection. **Panel A**. CK19 signal detected using quantitative real-time RT-PCR and expressed as cell equivalents of MDA-MB-361 from epithelial cell and mononuclear cell subfractions. **Panel B**. HER2 signal detected using quantitative real-time RT-PCR and expressed as cell equivalents of MDA-MB-361 from epithelial cell and mononuclear cell subfractions.

To better understand the source of HER2 and CK19 signal from peripheral blood we isolated subpopulations of mononuclear cells from blood of 4 normal donors for measurement of these markers. PBMC were isolated by gradient centrifugation and subjected to serial immunomagnetic positive selection with antibody against monocytes (CD14), lymphocytes (CD3/CD19), and natural killer cells/granulocytes (CD16). We found that CK19 signal was most commonly expressed in the lymphocyte population (CD3/CD19 population) (See Figure 4) A second experiment from a new group of four normal donors confirmed these findings showing that the lymphocyte population (CD3/CD19) contained the highest CK19 signal and the NK cells/granulocytes (CD16) population demonstrated the highest abundance of HER2 expression in all 4 new donors (data not shown). Isolation of natural killer cells using anti-CK56 antibody showed that these cells were also a source of HER2; however, expression of HER2 in this subpopulation varied by subject and was not the only source of signal in the CD16 fraction.

**Figure 5:**
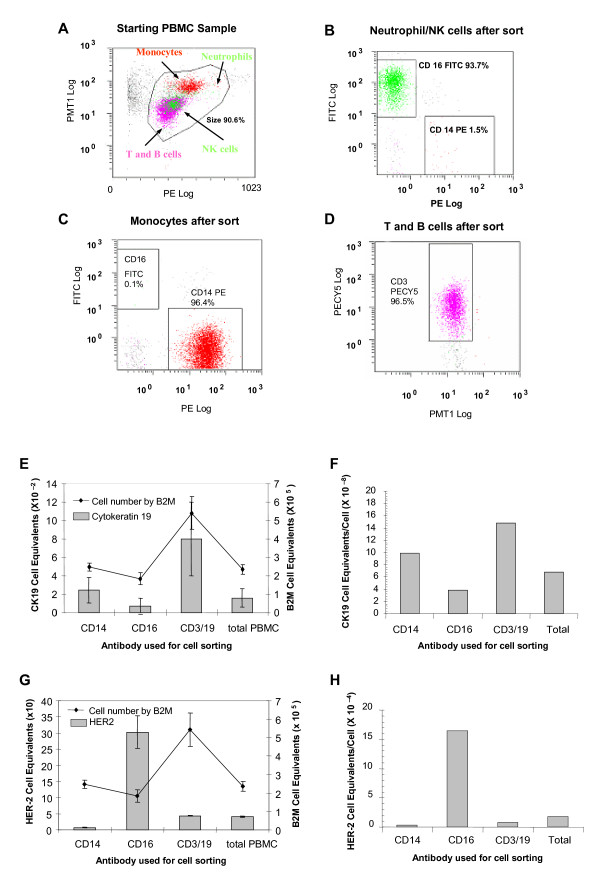
**A-D Flow cytometry sorting of peripheral blood mononuclear cells (PBMC) subpopulations: CD3/CD19, CD14, and CD16**. PBMC were incubated with an antibody cocktail of CD3-Cy5, CD19-APC, CD16-FITC, and CD14-PE, then placed in the cell sorter. This method showed 93.7%, 96.4% and 96.5% purity for CD16, CD14 and CD3/19 fractions respectively. **E-H. CK19 and HER2 signal was detected in sorted subpopulations of Peripheral Blood Mononuclear Cells**. RNA from subpopulations of flow cytometry sorted cells was isolated and subjected to quantitative RT-PCR for CK19, HER2, and B2M. HER2 and CK19 expression/cell was calculated as the ratio of HER2/CK19 cell equivalents over the total number of PBL cells within the sample (determined using B2M expression). **E**. CK19 expression in sorted normal PBL. **F**. CK19 expression/cell in sorted normal PBL. **G**. HER2 expression in sorted normal PBL. **H**. HER2 expression/cell in sorted normal PBL

To further confirm the sources of CK19 and HER2 signals in peripheral blood, PBMC from a normal donor were labeled with fluorescent conjugated antibodies to CD3/CD19 (Magenta), CD16 (green), and CD14 (red) and subjected to FACS analysis (See Figure 5A–D). This method was highly effective at purification of cellular subtypes with 93.7%, 96.4% and 96.5% purity for CD16, CD14 and CD3/19 fractions respectively. RNA from these subpopulations was isolated and subjected to quantitative RT-PCR for CK19 and HER2. These experiments again demonstrated that the CK19 signal was observed predominantly in the lymphocyte population (CD3/CD19), although some expression of CK19 in the monocyte (CD14) population was seen (See Figure 5E, F) Confirming the results of immunomagnetic selection experiments, HER2 expression was predominantly seen in the CD16 population (NK/granulocytes) (See Figure 5G.H).

HER2 expression per cell, based on the ratio of HER2 to B2M, was significantly higher than CK19 (CD3/CD19 500×, CD16 40,000×, CD14 300×). Therefore, it appears that fewer CD16 positive cells are required to generate background HER2 expression.

**Figure 6:**
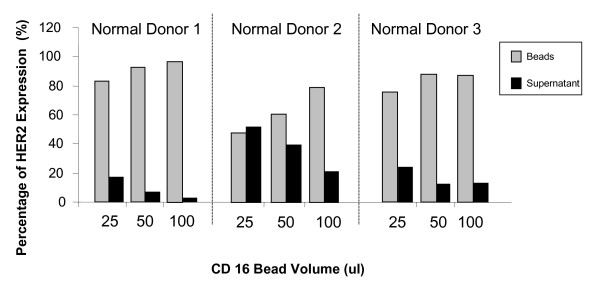
**Increasing volume of CD16 beads resulted in HER2 signal depletion**. Negative selection was performed on 3 PBL samples per 3 normal subjects with increasing amounts of CD16-labelled immunomagnetic beads. HER2 RNA expression was measured in both the CD16 selected cells (beads) and unselected cells (supernatant) by Real-time qRT-PCR. Data is expressed as the percent HER2 signal in each subfraction (bead or supernatant) over total HER2 signal for that subject.

In an attempt to deplete HER2 signal in peripheral blood, and improve the specificity of detection of HER2 overexpressing breast cancer cells, we performed negative selection with increasing amounts of α-CD16-labeled immunomagnetic beads using blood from three normal donors. This resulted in a dose-dependent depletion of HER2 mRNA from the supernatant, suggesting that this subpopulation was the source of the HER2 signal (See Figure 6) In addition, it appears that part of the HER2 signal can be removed from the peripheral blood, although baseline HER2 and the efficiency of HER2 selection varied by donor (74–98%). Therefore, negative selection for CD16 is one method whereby contaminating HER2 PBMC might be removed in studies of circulating cancer cells.

## Discussion

The study of circulating tumor cells is an important area of research with various clinical implications. Accumulated evidence suggests that CTC detected in the blood and DTC detected in the bone marrow of breast cancer patients are independent prognostic factors of disease free and overall survival [1,11-17]. The clinical impact of CTC in the blood and DTC in the bone marrow and the fact that CK19 positive cells present in the bone marrow were shown to have clonogenic potential suggest that these cells are unlikely to be benign 'innocent bystanders'[18].

The capacity to detect CTC in peripheral blood gives researchers non-invasive and more practical ways to use these markers in a wider clinical setting. However, technical challenges associated with detecting small numbers of malignant cells in the peripheral blood have limited the use of this approach. The development of ultra-sensitive molecular biological techniques has facilitated this very important area of research; however specificity issues remain a concern.

As with IHC, cytokeratins are most the frequently used targets to detect breast cancer cells in bone marrow or peripheral blood using RT-PCR. In serial dilution assays, RT-PCR detects CK expression from 1 tumor cell in 10^6 ^or 10^7 ^mononuclear cells [19-21]. However, PCR can be associated with false positive results – the most important limitation of this technique [5,22,23]. False positives are thought to result mainly from three sources: 1) amplification of pseudogenes from contaminating genomic DNA; 2) amplification of illegitimately transcribed genes by hematopoietic cells and 3) amplification of epithelial genes from contaminating non-tumor cells [24-27]. Researchers have shown that careful primer design can eliminate the first issue[28]. However, the other two sources of false positive results are difficult to deal with using a highly sensitive method such as RT-PCR. Quantitative, real-time RT-PCR allows quantitation of the transcript; therefore, differences in expression between normal and tumor cells may be better appreciated[29]. In addition, the quantitation of expression may allow assessment of expression levels of the target and provide additional information concerning the biology of the target being studied.

We identified the major source of CK19 in PBMC to be the lymphocyte population. Our experience also shows that it is possible to reduce CK19 background to a certain level (when the enrichment for CTC is over 1000-fold) by immunomagnetic selection and use this method to detect circulating tumor cells in clinical patients, with improved specificity.

Limited data exists on the expression of HER2 in micrometastatic cells. Braun, *et al*. have evaluated the presence of HER2 positive cells in the bone marrow of breast cancer patients by IHC or PCR. HER2 signal was positively correlated with a higher tumor stage but was not found to be associated with any established prognostic factors, including the expression of HER2 in the primary tumor[30]. Patients whose bone marrow cells demonstrated HER2 expression had a worse survival and HER2 expression in these cells was an independent prognostic factor. Although these results are intriguing, the population in this study was small. Furthermore, the discordance between expression in the bone marrow and the primary tumor is unexpected as HER2 expression is generally maintained in tumor cells throughout cancer progression and into the metastatic deposits[31]. Other investigators have attempted to measure HER2 in malignant cells in the circulation, and also report discordance between expression of HER2 in circulating cells compared with the primary tumor[6,32].

Our findings are consistent with the notion that white blood cells present in blood or bone marrow may be the source of false positive readings for HER2, and express this marker at an unexpectedly high per cell level in peripheral blood natural killer cell and granulocyte populations. While the expression of HER2 in normal PBMC may still be much lower than HER2 levels in malignant tumors that overexpress the gene, the relative frequency of malignant epithelial cells in the circulation is much lower than that of the mononuclear cells (1 per 10^5^–10^7^) making the background signal an important source of false positive results. In addition, the problem of background expression of HER2 in PBMC is more pronounced than that observed with CK19. The relative levels of HER2 are lower than CK19 in epithelial cells, (even in cells with an amplified HER2 gene) while the expression of HER2 is higher than the expression of CK19 in PBMC. The negative selection we used reduced the background HER2 to some extent, but is still not specific enough to be used in a clinical setting.

## Conclusion

In conclusion, we present a novel approach to improve the specificity of the established method to detect CTC by identifying the source of the background signals and reducing them by the proposed method of negative immunoselection. Our method was successful in reducing background CK19 signals, which will improve specificity in detecting CTC. However, based upon our experience, it is still premature to use HER2 as an RT-PCR marker for circulating tumor cells until the development of improved methods of negative and positive selection to remove the source of background signals from peripheral blood samples.

Non invasive and highly specific and sensitive methods of detecting CTC will prove to be extremely useful tools for clinicians in diagnosing breast cancers, determining prognosis and monitoring treatment responses. More effort should be invested in optimizing these methods.

## List of abbreviations

CTC: Circulating tumor cells; PBMC: Peripheral blood mononuclear cells; CK19: Cytokeratin; B2M: Beta 2 microglobulin; RT-PCR: Reverse transcription polymerase chain reaction

## Competing interests

Lisa A Roberts and Natalie A Solomon are employed by Abbott Molecular, Inc.

Paula N Friedman was employed by Abbott Molecular, Inc. at the time of the study.

Fanglei You, S. Peter Kang, Raquel A. Nunes, Cinara Dias, J. Dirk Iglehart and Lyndsay N. Harris declare that they have no competing interests

## Authors' contributions

FY Participated in the design of the study, carried out molecular studies, and drafted manuscript, LR Participated in the design of the study, carried out molecular studies, and drafted manuscript, SPK Reviewed draft and revised of the manuscript, RN Carried out molecular studies and reviewed manuscript, CD Carried out molecular studies, DI Participated in the design of the study and reviewed manuscript, NS Participated in the design of the study, involved in drafting and revision of the manuscript, PF Participated in the design of the study, interpretation of data, involved in drafting and revision of the manuscript, LNH Designed the study, involved in analysis and interpretation of data, drafted and revised manuscript. All authors read and approved the final manuscript
